# The effect of transcutaneous auricular vagus nerve stimulation on cardiovascular function in subarachnoid hemorrhage patients: a safety study

**DOI:** 10.1101/2024.04.03.24304759

**Published:** 2024-04-03

**Authors:** Gansheng Tan, Anna L. Huguenard, Kara M. Donovan, Phillip Demarest, Xiaoxuan Liu, Ziwei Li, Markus Adamek, Kory Lavine, Ananth K. Vellimana, Terrance T. Kummer, Joshua W. Osbun, Gregory J. Zipfel, Peter Brunner, Eric C. Leuthardt

**Affiliations:** 1 Department of Neurosurgery, Washington University School of Medicine, St. Louis, MO, USA; 2 Department of Biomedical Engineering, Washington University in St. Louis, MO, USA; 3 Department of Neuroscience, Washington University in St. Louis, MO, USA; 4 Department of Neurology, Washington University in St. Louis, MO, USA

**Keywords:** Subarachnoid hemorrhage, transcutaneous auricular vagus nerve stimulation, heart rate variability

## Abstract

**Objective:**

Subarachnoid hemorrhage (SAH) is characterized by intense central inflammation, leading to substantial post-hemorrhagic complications such as vasospasm and delayed cerebral ischemia.^2,6,7^ Given the anti-inflammatory effect of transcutaneous auricular vagus nerve stimulation (taVNS) and its ability to promote brain plasticity, taVNS has emerged as a promising therapeutic option for SAH patients.^3,8,9^ However, the effects of taVNS on cardiovascular dynamics in critically ill patients like those with SAH have not yet been investigated. Given the association between cardiac complications and elevated risk of poor clinical outcomes after SAH, it is essential to characterize the cardiovascular effects of taVNS to ensure this approach is safe in this fragile population^4^. Therefore, we assessed the impact of both acute taVNS and repetitive taVNS on cardiovascular function in this study.

**Methods:**

In this randomized clinical trial, 24 SAH patients were assigned to either a taVNS treatment or a Sham treatment group. During their stay in the intensive care unit, we monitored patient electrocardiogram (ECG) readings and vital signs. We compared long-term changes in heart rate, heart rate variability, QT interval, and blood pressure between the two groups. Additionally, we assessed the effects of acute taVNS by comparing cardiovascular metrics before, during, and after the intervention. We also explored rapidly responsive cardiovascular biomarkers in patients exhibiting clinical improvement.

**Results:**

We found that repetitive taVNS did not significantly alter heart rate, corrected QT interval, blood pressure, or intracranial pressure. However, taVNS increased overall heart rate variability and parasympathetic activity from 5–10 days after initial treatment, as compared to the sham treatment. Acutely, taVNS increased heart rate, blood pressure, and peripheral perfusion index without affecting the corrected QT interval, intracranial pressure, or heart rate variability. The acute post-treatment elevation in heart rate was more pronounced in patients who experienced a decrease of more than 1 point in their Modified Rankin Score at the time of discharge.

**Conclusions:**

Our study found that taVNS treatment did not induce adverse cardiovascular effects, such as bradycardia or QT prolongation, supporting its development as a safe immunomodulatory treatment approach for SAH patients. The observed acute increase in heart rate after taVNS treatment may serve as a biomarker for SAH patients who could derive greater benefit from this treatment.

## 1. Introduction

Subarachnoid hemorrhage (SAH) is a devastating subtype of stroke that represents a significant global health burden and causes permanent disability in approximately 30% of survivors.^1,5,32^ SAH is characterized by intense central inflammation leading to substantial post-hemorrhagic complications such as vasospasm and delayed cerebral ischemia, which are major contributors to post-SAH mortality and morbidity.^2,6,7^ In the quest to mitigate these inflammatory-driven complications and improve recovery from SAH, transcutaneous auricular vagus nerve stimulation (taVNS) has emerged as a promising therapeutic option. This is attributed to its anti-inflammatory effect and its ability to enhance brain plasticity.^3,8,9^

**Figure 1.**
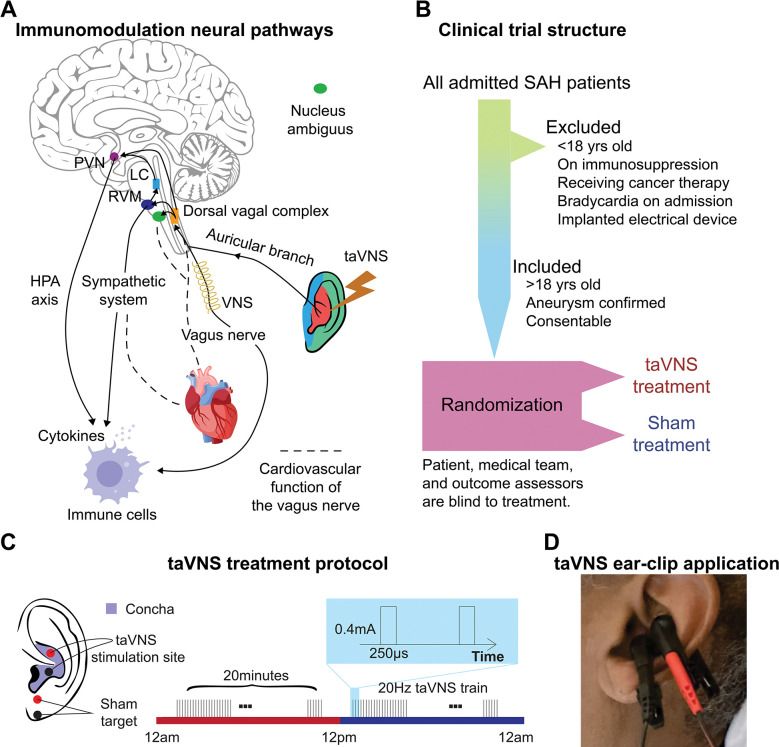
Study rationale and clinical trial design. **A.** Immunomodulation neural pathways associated with vagus nerve stimulation include cholinergic anti-inflammatory pathway, sympathetic nervous system, and hypothalamic-pituitary-adrenal (HPA) axis. Immunogenic stimuli activate vagal afferents terminating primarily in the dorsal vagal complex. Ascending projections from the dorsal vagal complex reach the paraventricular nucleus (PVN) and rostral ventromedial medulla (RVM), activating the hypothalamic-pituitary-adrenal (HPA) axis and sympathetic system, respectively, to regulate the immune response. taVNS can affect cardiovascular function through the sympathetic system or efferent vagus nerve. **B**-**C**. Clinical trial structure and treatment protocol. **D.** Ear clip application for taVNS treatment.

The auricular branch of the vagus nerve is a sensory nerve that innervates the external ear, including the cymba concha. Stimulating the auricular branch of the vagus nerve has been shown to activate the same brain regions as cervical vagus nerve stimulation^11^. Prior studies have shown that vagus nerve activity mediates cholinergic signaling and regulates proinflammatory responses via the inflammatory reflex (Figure 1A).^12,13^ Inflammatory mediators, such as cytokines, activate afferent vagus nerve signaling, which leads to efferent vagus nerve activation that reduces the production of pro-inflammatory cytokines.^14^ Recent studies suggest that the inflammatory reflex may represent a therapeutic target for the treatment of central nervous system inflammatory diseases as well as suppressing inflammation in peripheral tissues and organs.^14^

The vagus nerve also mediates cardiovascular function by regulating the autonomic system and metabolic homeostasis (Figure 1A).^16^ Animal studies have reported a potential risk of bradycardia and decreased blood pressure associated with vagus nerve stimulation^15^. Given the premise that taVNS mediates cardiovascular function, it is essential to thoroughly examine its cardiovascular implications in critically ill patients, such as those with SAH, to ensure its safety in this fragile population. However, our limited understanding of these effects constitutes a significant barrier, preventing the advancement of taVNS from a promising therapeutic approach to an established clinical treatment for SAH. This is particularly notable as cardiac complications following SAH are associated with an increased risk of poor outcomes^4^. This study aimed to assess the effects of taVNS on cardiovascular function based on electrocardiogram (ECG) and other monitored vital signs from SAH patients receiving treatment in the intensive care unit (ICU). The primary outcome metrics examined in this study include heart rate, heart rate variability, QT interval, and blood pressure. As parasympathetic innervation of the heart is partially controlled by the vagus nerve, we hypothesized that taVNS would increase heart rate variability without significantly affecting heart rate, QT interval, or blood pressure. Additionally, we sought to identify potential biomarkers that indicate which SAH patients may experience the most clinical benefit from taVNS therapy.

## 2. Methods

### 2.1 Study Participants

Participants in this study were recruited from adult patients who were admitted to the ICU at Barnes Jewish Hospital, St. Louis, MO, following an acute, spontaneous, aneurysmal SAH. Inclusion criteria were: (**1**) Patients with SAH confirmed by CT scan; (**2**) Age > 18; (**3**) Patients or their legally authorized representative are able to give consent. Exclusion criteria were: (**1**) Age < 18; (**2**) Use of immunosuppressive medications; (**3**) Receiving ongoing cancer therapy; (**4**) Implanted electrical device; (**5**) Bradycardia on admission; (**6**) Considered moribund/at risk of imminent death. Participants were randomized to receive either the taVNS (N = 11) or Sham (N = 13) treatment. Patients were enrolled prior to randomization by a member of the research team who went through the informed consent process with the patient or their legally authorized representative. Treatment group assignment was via a computer-generated randomization sequence, with the next number obscured until patient enrollment. Research team members who applied the ear clips and set stimulation parameters were not blinded to the treatment. The participants, the medical team who dictated all management decisions for the patient’s subarachnoid hemorrhage, and the outcomes assessors who assigned modified Rankin Scores (mRS) at admission and discharge were blinded to the treatment. The structure of this study is shown in Figure 1B. This study was approved by the Washington University School of Medicine Review Board and was conducted in accordance with institutional and national ethics guidelines and the Declaration of Helsinki (Clinical trial number: NCT04557618).

### 2.2 taVNS protocol

Following randomization, enrolled patients underwent 20 minutes of either taVNS or sham stimulation twice daily during their stay in the ICU. During treatment periods, a portable transcutaneous electrical nerve stimulation (TENS) device (TENS 7000 Digital TENS Unit, Compass Health Brands, OH, USA) was connected to the patient’s left ear using two ear clips (Figure 1C and D). For taVNS treatments, these ear clips were placed along the concha of the ear, while for sham treatments, the clips were placed along the earlobe to avoid stimulation of the auricular vagus nerve from tactile pressure (Figure 1Figure 1C). For the taVNS group, stimulation parameters were selected based on values reported in prior studies that sought to maximize vagus somatosensory evoked potentials while avoiding the perception of pain: 20 Hz frequency, 250 μs pulse width, and 0.4 mA intensity^17^. No electrical current was delivered during sham treatments. For both groups, the TENS device was connected to the patient and a bedside recording computer. The computer recorded continuous ECG and vital signs, including blood pressure, temperature, respiration rate, peripheral perfusion index, intracranial pressure, and arterial blood pressure. The collection of intracranial pressure and arterial blood pressure data varied, being dependent on the treatment protocol assigned by the clinical team, and thus was not uniformly available for all patients throughout the study. Please see ^18^ for a detailed protocol of this study.

### 2.3 Data processing

A 3-lead system was used for electrocardiograms (ECG). ECG signals, sampled at 500 Hz, and other vital signs, such as blood pressure, sampled at 1 Hz, were recorded from the Intellivue patient monitor (Philips^®^, Netherlands) using vitalDB software.^19^

**Figure 2.**
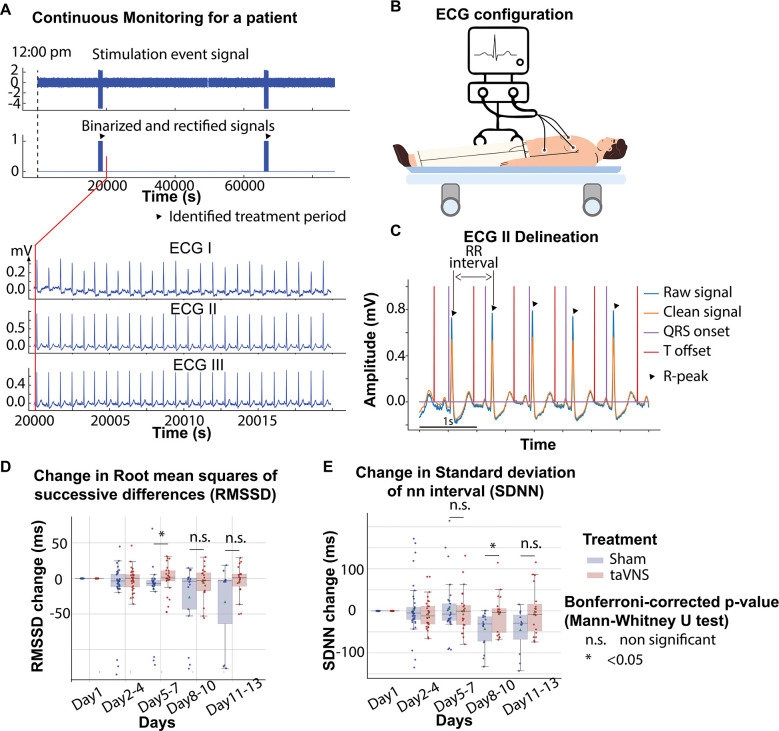
The effects of taVNS on cardiac function. **A**. Signals encoding treatment period and ECG signals in a representative patient. **B**. 3-lead ECG configuration in the intensive care unit. **C**. P wave, T wave, and QRS complex are delineated from clean ECG II signals. **D and E**. The standard deviation of NN interval (SDNN) changes and Root mean squares of successive differences change over time for the two treatment groups. The color represents the treatment group. P-values were calculated from Mann–Whitney U tests and were corrected based on the Bonferroni method. Green triangles represent the mean.

To calculate cardiac metrics, we first applied a 0.5 Hz fifth-order high-pass Butterworth filter and a 50 Hz powerline filter on ECG data to reduce artifacts. We detected QRS complexes based on the steepness of the absolute gradient of the ECG signal using Neurokit2 software package.^20^ R-peaks were detected as local maxima in the QRS complexes. P-waves, T-waves, and QRS waves were delineated based on the wavelet transform (Figure 2A–C).^21^ RR intervals were preprocessed to exclude outliers, defined as RR intervals greater than 2 s or less than 300 ms. RR intervals with > 20% relative difference to the previous interval were considered ectopic beats and excluded from analyses. After preprocessing, RR intervals were used to calculate heart rate, heart rate variability, and corrected QT based on Bazett’s formula.^33^ Heart rate variability measures included the root mean square of successive difference of normal RR intervals (RMSSD), which indicates parasympathetic activity, and the standard deviation of normal RR intervals (SDNN), which is a clinical measure of cardiac risk.^22,23^ Heart rate variability calculations are detailed in Supplementary Materials.

To investigate the effect of repetitive taVNS on cardiovascular function, we compared heart rate variability, heart rate, corrected QT intervals, blood pressure, and intracranial pressure calculated over 24 hours between patients receiving taVNS and sham treatment. In addition, we compared the mean peripheral perfusion index and respiration rate over 24 hours between treatment groups to determine the effects of repetitive taVNS on the autonomic system. Data collection commenced on the first day of each patient’s ICU admission. The average duration of continuous data recording was 11.1 days, with a standard deviation of 6.8 days. To analyze the effects of taVNS treatment more granularly, we segmented the changes in these metrics from the initial day at three-day intervals, facilitating comparison between the taVNS and sham treatment groups over the course of their ICU stay.

To study the effects of acute treatment over time, we focused on blood pressure, heart rate variability, heart rate, and corrected QT intervals 20 minutes before treatment (pre-treatment), during the 20-minute treatment (during-treatment), and 20 minutes after treatment (post-treatment). The treatment event signals were rectified and binarized based on their half-maximum value to identify the treatment onset and offset (Figure 2A). We calculated metrics using 6-minute sliding windows over ECG data starting from treatment onset/offset and moving bi-directionally with a 3-minute step. To correct daily and between-subject variation, we applied the same sliding window strategy to calculate the mean and standard deviation of these cardiac metrics for each patient for each day as a reference. Subsequently, heart rate variability, heart rate, and corrected QT interval around treatment onset/offset were normalized based on the reference. In addition, we calculated the difference in blood pressure, heart rate variability, heart rate, and corrected QT intervals between during-treatment and pre-treatment, and the difference between post-treatment and pre-treatment for each patient and for each treatment. To study the effects of acute taVNS, we compared the two differences between the treatment groups.

### 2.4 Factor Analysis

We performed an exploratory factor analysis to identify the factors underlying autonomic system activity. Besides RMSSD and SDNN, variables derived from preprocessed RR intervals and used to perform factor analysis included the percentage of successive normal-to-normal (NN) Intervals that differ by more than 50 ms (pNNI_50), total power (below 0.4 Hz), normalized high-frequency power (0.15–0.4Hz), cardiac vagal index, and cardiac sympathetic index. The total power is thought to represent the overall heart rate variability, while normalized high-frequency power primarily reflects parasympathetic activity^22^. These variables were normalized using a z-score method based on individual daily means and standard deviations before factor analysis. Factor analysis was performed using the factor_analyzer Python package. The number of factors was set to 2 based on the Scree plot. The factor loadings were calculated using the Minimum Residual Method. After factor extraction, a Varimax rotation was applied for better interpretability, so that each factor has high loadings for a smaller number of variables and low loadings for the remaining variables.

### 2.5 Statistical Analyses

We grouped the change of heart rate variability, heart rate, and corrected QT interval from the first hospitalized day in bins of three days and compared them between treatment groups using Mann–Whitney U tests. The change in blood pressure, intracranial pressure, respiration rate, and peripheral perfusion index from the first hospitalization day were also compared using Mann–Whitney U tests. We used Wilcoxon signed-rank tests to compare the difference in heart rate between post-treatment and during-treatment against 0. Power analysis was performed based on the t-test, assuming the medium effect size (Cohen’s d = 0.5). Two one-sided tests were used to confirm that taVNS did not induce long-term changes in heart rate, corrected QT interval, or blood pressure, with equivalency test margins set to 5 bpm for heart rate, 50 ms for QT interval, and 2 mmHg for blood pressure. A summary of statistical tests is provided in Supplementary Table 2.

## 3. Results

### 3.1 Cumulative effects of taVNS on cardiac function

We first studied the effect of taVNS on the autonomic system through heart rate variability. This is important because sympathetic dominance of the autonomic system is believed to play a key role in the development of cerebral vasospasm and cardiac aberrations following SAH.^24^ Figure 2D shows the changes in RMSSD of taVNS and Sham treatment groups over the course of treatment using Day 1 as the baseline. We compared RMSSD between groups after 4 days of treatment and found that RMSSD was significantly higher in the taVNS treatment group between Days 5–8 (Mann–Whitney U test, N(taVNS) = 25, N(Sham) = 29, Bonferroni-corrected p = 0.03, Cohen’s d =0.41, W-statistics = 510). We further tested if the RMSSD reduction rate was greater in the Sham treatment group with a linear regression model: RMSSD change ~ Day * Treatment. The results show that the RMSSD reduced slower in the taVNS treatment group when compared to taVNS, but this trend did not reach significance (coefficient of taVNS * Day interaction effect = 2.00, p = 0.21, Supplementary Figure 1). We also compared SDNN between groups and found that SDNN was significantly higher in the taVNS treatment group during Days 8–10, as compared to the Sham group (Mann–Whitney U test, N(taVNS) = 20, N(Sham)=17, Bonferroni-corrected p-value = 0.03, Cohen’s d =0.82, W-statistics = 253).

**Figure 3.**
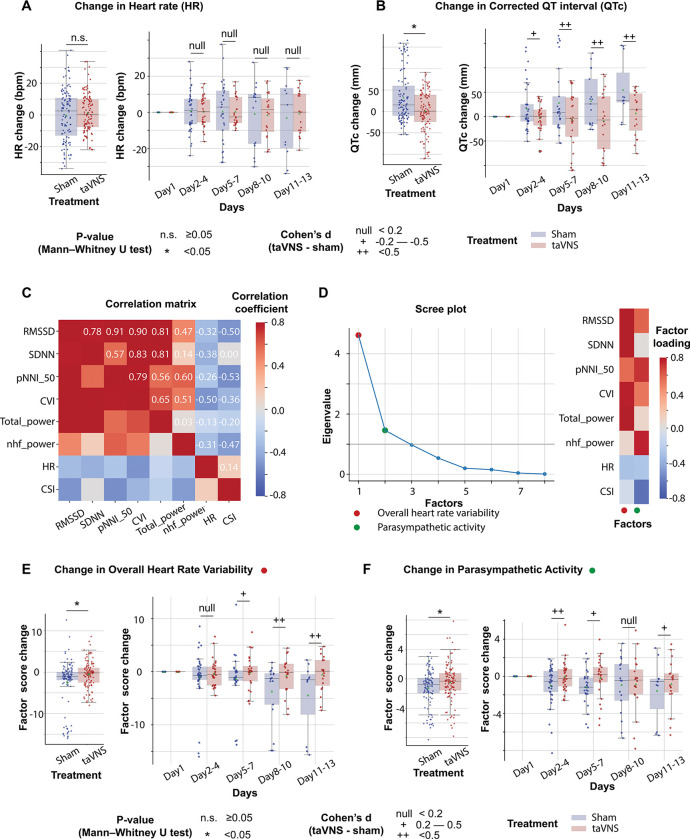
The effects of taVNS on overall heart rate variability and parasympathetic activity. **A-B**. Heart rate and corrected QT interval changes from the first hospitalized day in the two treatment groups. **C**. Correlation between standard ECG features underlying autonomic activities. **D**. Factor analysis showed that there are two factors underlying the standard ECG features. The first factor is referred to as overall heart rate variability. The second factor is referred to as parasympathetic activity. **E-F**. The effect of taVNS on the two factors. pNNI_50: Percentage of Number of successive NN Intervals that differ by more than 50 ms. CVI: cardiac vagal index. Total power: total power below 0.4Hz of normal RR interval. nhf_power: relative power of the high-frequency band (0.15–0.4 Hz). CSI: cardiac sympathetic index.

Subsequently, we investigated whether taVNS treatment induced bradycardia or QT prolongation, both potential adverse effects of vagus nerve stimulation. This analysis showed no significant differences in heart rate calculated from 24-hour ECG recording between groups (Mann–Whitney U test, N(taVNS) = 94, N(Sham)=95, p-value = 0.70, Cohen’s d =−0.01, W-statistics = 4317, power = 0.93). Change in heart rate from Day 1 was equivalent between groups (Two-tailed equivalence tests, *p*[*lower threshold*] = 0.006, test statistics[*lower threshold*] = 2.53; *p*[*lower threshold*] = 0.004, test statistics[*lower threshold*] = −2.72, N(VNS)=94, N(VNS)=95). We further confirmed that heart rate was similar between treatment groups over the course of treatment (Figure 3A |Cohen’s d| < 0.2 for Day 2–4, Day 5–8, Day 8–10, and Day 11–13). Moreover, the corrected QT interval was higher in the Sham group compared to the taVNS group (Figure 3B, Mann–Whitney U test, N(taVNS) = 94, N(Sham)=95, p-value = 0.01, Cohen’s d =−0.57). From day 5 to 13, the mean and median corrected QT interval were lower in the taVNS group with large effect sizes (Figure 3B, |Cohen’s d| > 0.5).

Figure 3C shows the correlation matrix of commonly used heart rate variability metrics. Bartlett’s test p-value of the correlation matrix is less than 0.01, indicating that there are significant relationships among these variables. Therefore, we used factor analysis to identify the underlying factors. We focused on the two factors with the greatest eigenvalue. Figure 3D shows the factor loading, that is, the variance explained by heart rate variability metrics on the two factors. The first factor correlates positively with metrics representing variability, including RMSSD, SDNN, pNNI_50, and total power, and therefore has been termed Overall Heart Rate Variability. The second factor correlates positively with RMSSD and normalized high-frequency power that represents parasympathetic activity, and correlates negatively with the cardiac sympathetic index. Hence, it is termed Parasympathetic Activity. The effects of taVNS on Overall Heart Rate Variability and Parasympathetic Activity aligned with the observed effects on SDNN and RMSSD. Overall, Heart Rate Variability change from Day 1 was significantly higher in the VNS group (Figure 3E, Mann–Whitney U test, N(taVNS) = 94, N(Sham)=95, p-value = 0.04, Cohen’s d =0.37). The effect size was trivial between Day 2–4 and increased over the course of treatment. The parasympathetic activity was also significantly higher in the VNS treatment group, and we observed the largest effect size between Day 2–4 (Figure 3F).

We also investigated the potential association between clinical outcomes, as measured by changes in the mRS from admission to discharge, and heart rate variability metrics. We found that heart rate was lower in patients with improved mRS (i.e., <0) (Mann–Whitney U test, N(mRS < 0) = 122, N(mRS > 0)=98, p-value < 0.01, Cohen’s d =−0.54). Parasympathetic Activity, Overall Heart Rate Variability, and corrected QT interval did not differ significantly between patients with improved mRS and patients with worsened mRS (Supplementary Figure 3).

### 3.2 Cumulative effects of taVNS on vascular function

**Figure 4.**
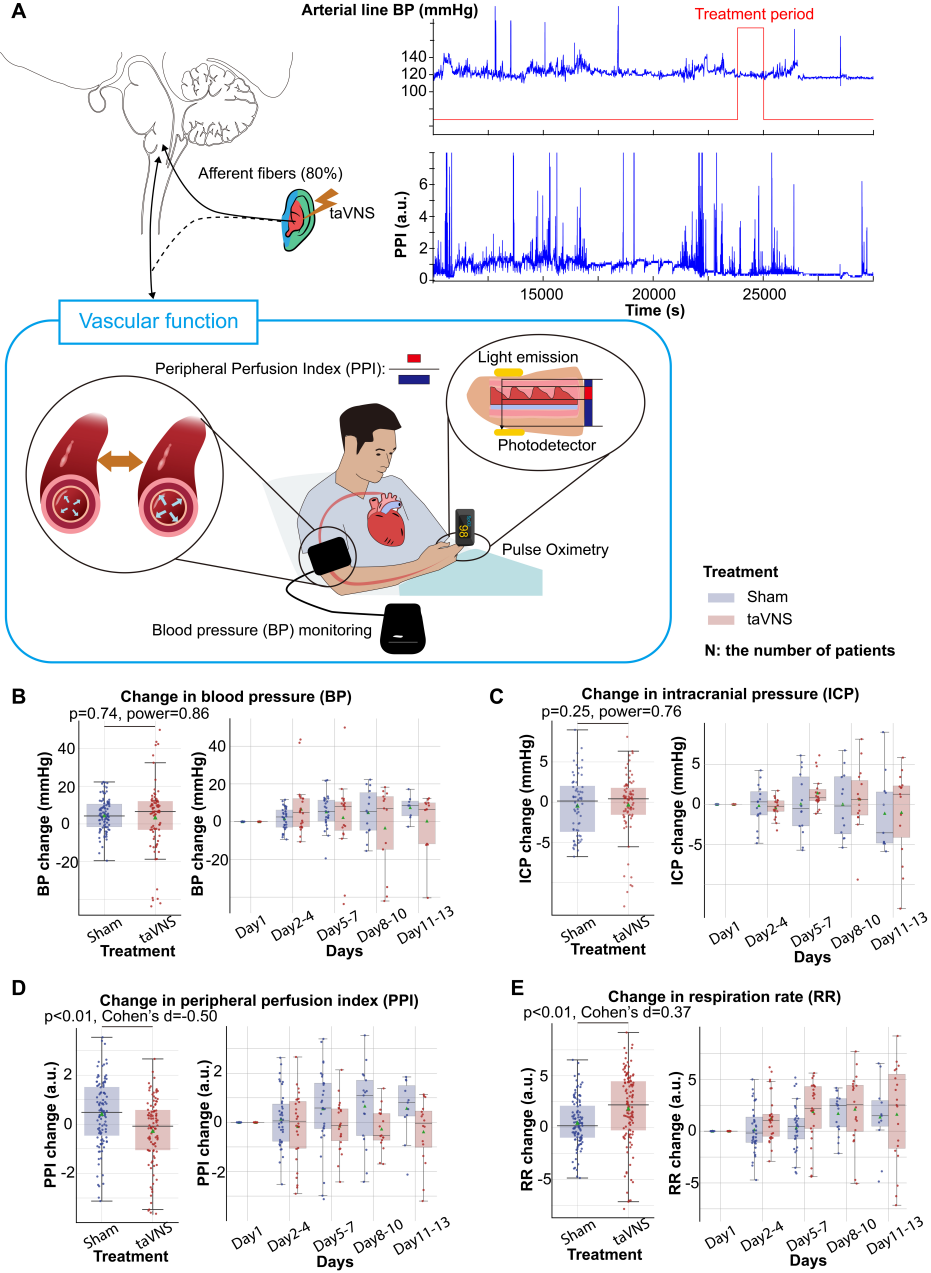
The cumulative effects of taVNS on vascular function. **A**. Representative vital signs and their physiology. Arterial line blood pressure (see supplementary Figure 4), intracranial pressure, and blood pressure measured regularly by nurses (NIBP) were recorded. Blood pressure is an index of vasodilation. PPI is the ratio between the pulsatile and the non-pulsatile blood flow, reflecting the cardiac output. **B-C.** NIBP and ICP changes from the first hospitalization day did not differ significantly between the treatment groups. **D-E**. PPI change from the first hospitalized day was lower in the VNS treatment group, while RR change was higher.

Elevated blood pressure is a common occurrence in SAH and is linked with a higher risk of re-rupture of cerebral aneurysms and vasospasm.^25,26^ Thus, we tested if blood pressure changes over the course of treatment were different between treatment groups. The median and mean blood pressure change from the first hospitalized day were greater than 0 for both treatment groups (Figure 4B). No significant differences were detected in changes in blood pressure and intracranial pressure (ICP) between the treatment groups (Figure 4B and C). Equivalence testing confirmed that the ICP changes from the first hospitalization day were not significantly different between treatment groups, with a 2mmHg equivalence margin (two one-sided t-tests, *p*[*lower threshold*] = 3.66 × 10^−13^, t[*lower threshold*] = 8.07; *p*[*upper threshold*] = 3.33 × 10^−10^, t[*upper threshold*] = −6.73). Equivalence testing also indicated that there were no significant different changes in blood pressure between treatment groups (two one-sided t-tests, *p*[*lower threshold*] = 0.07, t[*lower threshold*] = 1.51; *p*[*upper threshold*] = 0.002, t[upper threshold] = −3.00). We further verified that there were no significant changes in arterial line blood pressure obtained via continuous invasive monitoring between treatment groups (Supplementary Figure 4). We subsequently compared the Plethysmography Peripheral Perfusion Index (PPI) between the treatment groups as it is used as a proxy metric for cardiac stroke volume and vascular tone.^27,30^ We found that PPI change was significantly lower (Mann–Whitney U test, N(taVNS) = 83, N(Sham)=95, Bonferroni corrected p < 0.01, Cohen’s d =−0.49) while respiration rate change was significantly higher (Mann–Whitney U test, N(taVNS) = 94, N(Sham)=95, Bonferroni corrected p = 0.02, Cohen’s d =0.37) in the taVNS group, as compared to the Sham group (Figure 4D and E).

### 3.3 Acute effects of taVNS on cardiovascular function

**Figure 5.**
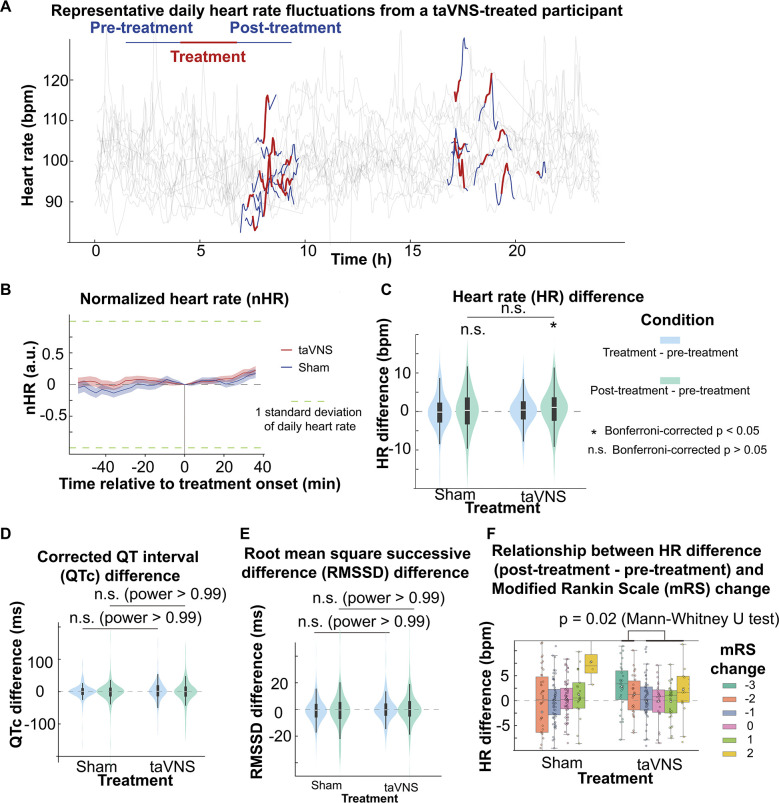
The acute effects of taVNS on cardiac function. **A**. Daily fluctuation of heart rate of a subject receiving VNS treatment. The treatment period, a 20-minute period before and after treatment, is highlighted. Note that a small proportion of ECG signals to derive heart rate was missing due to the expected cyclical restarting of the monitoring system. **B**. Normalized heart rate aligned at the treatment onset over time for the two treatment groups. The heart rate is normalized based on the mean and standard error of heart rate for each day. **C-D**. The difference of HR, QTc, and RMSSD between the treatment period, post-treatment period, and pre-treatment period for the two groups. Wilcoxon signed ranked test was used to test if the HR difference is statistically different from 0 in the VNS treatment group. Bonferroni-corrected p-value for HR difference between post-treatment and treatment period is 0.03 (N=188, Cohen’s d = 0.1). Mann–Whitney U tests were used to compare cardiac function metric differences between the two treatment groups. **F**. Heart rate changes following acute taVNS in patients grouped based on treatment and mRS change.

Understanding the acute effect of taVNS on cardiovascular function is an essential step to translating it towards clinical usage. We compared the acute change of heart rate, corrected QT interval, and heart rate variability between treatment groups as these metrics indicate cardiovascular complications and predict clinical outcomes.^28^ The change in heart rate from treatment onset is shown in Figure 5B. We subsequently tested whether there was a difference in heart rate between post-treatment and pre-treatment for both groups. We found that heart rate significantly increased in the taVNS group (Wilcoxon rank-sum test, N = 188, Bonferroni corrected p = 0.03, Cohen’s d =0.11) but not in the Sham group (Wilcoxon signed ranked test, N = 199, Bonferroni corrected p = 0.72, Cohen’s d =0.00) (Figure 5C). However, the increase in heart rate was not significantly different between treatment groups, and the increase in heart rate after taVNS was within 0.5 standard deviations of daily heart rate, making the statistical difference not clinically meaningful. There were no significant differences in changes in corrected QT interval or heart rate variability, as measured by RMSSD and SDNN, between treatment groups (Figure 5D and E and Supplementary Figure 5). We further asked whether heart rate can serve as a biomarker that indicates which SAH patients would receive the greatest benefit from continuing taVNS treatment. We investigated the relationship between changes in heart rate from pre- to post-taVNS treatment and changes in mRS between admission and discharge. We found significantly greater increases in heart rate were induced following taVNS treatment in patients who had an improvement in mRS of −2 or greater compared to other patients (Mann-Whitney U test, p=0.02, N(mRS change <−1) = 53, N(mRS change >= −1) = 135, Cohen’s d=0.34, Figure 5). Conversely, acute heart rate variability change, represented by RMSSD, was not significantly different based on mRS in SAH patients (Supplementary Figure 5).

Subsequently, we compared changes in blood pressure, PPI, ICP, and respiration rate from pre- to post-treatment periods between treatment groups. We found that changes in PPI and blood pressure were significantly higher in the taVNS group, as compared to the Sham group (Mann-Whitney U test, blood pressure: *p* = 0.03, Cohen’s d = 0.22, N = 180 for Sham and 159 for taVNS; PPI: *p* < 0.01, Cohen’s d = 0.19, N = 227 for Sham and 186 for taVNS, Supplementary Figure 6). Only PPI remained significantly different between treatment groups after Bonferroni correction. The acute changes in PPI and blood pressure remained within the daily standard deviation. No significant differences in post-treatment changes in ICP or respiration rate were observed between treatment groups.

## Discussion

This study examined the effects of transcutaneous auricular vagus nerve stimulation (taVNS) on cardiovascular function in patients with subarachnoid hemorrhage (SAH). We investigated both the cumulative and acute impacts of taVNS. The findings in our study indicates that repetitive taVNS is not associated with previously suggested risks, such as bradycardia and QT prolongation. Furthermore, cumulative taVNS treatment increased overall heart rate variability and parasympathetic activity, which are indicators of a healthy cardiovascular system. When looking at the acute effects, taVNS only significantly increased the peripheral perfusion index but not heart rate, heart rate variability, corrected QT interval, blood pressure, or intracranial pressure. The findings are summarized in Table 1. Interestingly, we discovered that heart rate as a biomarker for identifying SAH patients who are most likely to benefit from taVNS treatment. Collectively, this study substantiates the safety of treating SAH patients with taVNS and provides foundational data for future efforts to optimize and translate taVNS therapy toward clinical use.

### taVNS and autonomic system

The autonomic nervous system (ANS), comprising the sympathetic nervous system and the parasympathetic nervous system, plays a critical role in maintaining physiological homeostasis. These systems work synergistically to mediate interactions between the nervous and immune systems, which is thought to be the underlying mechanism for the immunomodulatory effect of taVNS. Our study is aligned with the finding that the autonomic balance is shifted toward sympathetic dominance following SAH (Figure 3, Supplementary Figure 2). In this study, we found that dysregulation of sympathovagal balance toward sympathetic dominance could be restored by taVNS treatment.^29,34^

A key metric that reflects this restored sympathovagal balance is the increase in heart rate variability. That said, there are more complex interactions that will require further investigation. Specifically, the interactions between Plethysmography Peripheral Perfusion Index (PPI) and blood pressure are more nuanced. PPI measures are primarily influenced by cardiac output and vascular tone. Elevated PPI is associated with vasodilation and/or increased stroke volume. Increased blood pressure is associated with vasoconstriction and/or increased stroke volume.^30^ In the Sham group, increases in both PPI and blood pressure were observed when compared to Day 1 values (Figure 3). This effect may be due to higher stroke volume resulting from sympathetic activation following SAH and/or could represent the heightened need for vasopressor interventions aiming to improve cerebral perfusion due to more robust sympathetically driven cerebral vasospasm. For the taVNS group, however, while the blood pressure change over days was similar between groups, the PPI increase was reduced (Figure 4). This would suggest elevated vasoconstriction, which is counterintuitive, given the notion of taVNS enhancing vagal tone. One possible explanation for this observation could be that taVNS-induced parasympathetic activation reduced cardiac stroke volume and activated compensatory mechanisms to maintain blood pressure by increasing peripheral resistance. Additional considerations are the differences between repetitive and acute changes associated with taVNS. Although repetitive taVNS increases heart rate variability days after initiation of the treatment, this effect is not seen acutely. Also, while repetitive taVNS was associated with a reduced PPI and no change in heart rate and blood pressure, there were small acute increases in PPI, heart rate, and blood pressure. These results indicate that taVNS might increase arousal in the acute period.^36,37^ Ultimately, these speculative mechanisms warrant further validation through animal or pharmacological studies directly investigating the effects of taVNS on autonomic function and vascular tone.

### Considerations for applying taVNS on SAH patients

Blood pressure management and cardiac function monitoring are crucial in patients following SAH.^25^ This study shows that blood pressure, QT interval, and heart rate remained similar in both treatment groups. This suggests that treating SAH patients with taVNS is unlikely to cause adverse blood pressure alterations or cardiac complications. Our findings also suggest that repetitive taVNS could rescue the sympathetic excitation and parasympathetic withdrawal following SAH, which could lead to favorable clinical outcomes.^34^ Additionally, increased heart rate variability, reduced PPI, and increased respiration rate were observed after repetitive taVNS, suggesting that the autonomic system self-regulates to maintain cardiovascular homeostasis. Thus, it is important to consider autonomic system self-regulation when studying the therapeutic effects of taVNS.^31^ Also, it is important to note that while acute cardiovascular changes were noted after taVNS, these changes were within normal daily variations in this study, making them unlikely to pose a risk to the patient. That said, the observed acute increases in PPI following taVNS necessitate caution when considering taVNS treatment for patients to whom peripheral vasodilatation is not desired.

### Limitations and outlook

While this study supports the safety of taVNS treatment in SAH patients, the findings may not be universally applicable across different patient populations. For instance, a decrease in heart rate variability was noted in the Sham group, highlighting the influence of SAH pathology on cardiovascular metrics. Importantly, additional care should be paid when interpreting the results of blood pressure, as hypertension was intentionally induced for some patients being treated for vasospasm. Patient medical histories are summarized in Supplementary Table 1.

We note that heart rate was lower in patients with improved mRS, while other cardiovascular metrics were not different between patients with improved mRS and patients with worsened mRS (Supplementary Figure 3 and Supplementary Figure 7). Future studies should aim to delineate the relationship between cardiovascular function, the autonomic system, and SAH complications, such as vasospasm, to rigorously assess the efficacy of taVNS treatment.

## Conclusions

Utilizing taVNS as a neuromodulation technique in SAH patients appears to be safe without inducing bradycardia or QT prolongation. Repetitive taVNS treatment increased parasympathetic activity. Acute taVNS elevated heart rate, which might be an acute biomarker to identify SAH patients who are likely to respond favorably to taVNS treatment.

## Supplementary Material

Supplement 1

## Figures and Tables

**Table 1.** Summary of effects of acute and repetitive taVNS on cardiovascular function in SAH patients.

		Positive findings	Null findings	Implications
**Repetitive**	Cardiac function	Increased HRV	QT interval Heart rate	Increased parasympathetic activity
	Vascular function	Reduced peripheral perfusion index Increased respiration rate	Blood pressure Intracranial pressure	Compensatory mechanisms to maintain autonomic balance
**Acute**	Cardiac function		Heart rate Heart rate variability QT interval	No to small acute effect
	Vascular function	Increased peripheral perfusion index	Intracranial pressure Respiration rate	

Metrics for cardiovascular function include heart rate variability, heart rate, QT interval, blood pressure, intracranial pressure, peripheral perfusion index, and respiration rate.

## Highlights

- Transcutaneous auricular vagus nerve stimulation (taVNS) treatment is a safe immunomodulatory treatment approach for subarachnoid hemorrhage patients.
- taVNS treatment did not induce adverse cardiovascular effects, such as bradycardia or QT prolongation.
- An acute increase in heart rate after taVNS treatment may serve as a biomarker for SAH patients who could derive greater benefit from this treatment.
