# Supplementary material for: The effect of transcutaneous auricular vagus nerve stimulation on cardiovascular function in subarachnoid hemorrhage patients: a safety study
Source: medRxiv. 2024 Apr 3:2024.04.03.24304759. Preprint. [Version 1] doi: 10.1101/2024.04.03.24304759 (PMC11023641; doi:10.1101/2024.04.03.24304759)
Supplement: Supplementary file 1 [file media-1.pdf]

Supplementary materials for: The effect of transcutaneous auricular vagus nerve stimulation on cardiovascular function in subarachnoid hemorrhage patients: a safety study

Gansheng Tan, Anna Huguenard, Kara M. Donovan, Phillip Demarest, Xiaoxuan Liu, Ziwei Li, Markus Adamek, Kory Lavine, Ananth K. Vellimana, Terrance T. Kummer, Joshua W. Osbun, Gregory J. Zipfel, Peter Brunner, Eric C. Leuthardt

*Correspondence to leuthardte@wustl.edu

Calculation of cardiovascular metrics

1. Heart rate (HR) over a period is the mean of $\frac{60}{NN\ interval\ in\ seconds}$ over this period.
2. Root mean square of successive difference of normal RR intervals (RMSSD) for a given period:

   $RMSSD\ = \sqrt{\frac{1}{N-1}\sum_{i}^{N-1}(NN_{i+1} - NN_i)^2}$, where N is the number of NN intervals within this period.
3. Standard deviation of normal RR intervals (SDNN) for a given period:

   $SDNN\ = \sqrt{\frac{1}{N}\sum_{i}^{N}(NN_i - \overline{NN})^2}$, where $\overline{NN}$ represents the mean of NN intervals within this period.

4. To calculate total power and normalized high-frequency power, power spectral density is calculated from NN intervals with the help of Python package *hrv-analysis*. Linear interpolation is used to make NN intervals evenly spaced, assuming a sampling frequency of 4Hz. Welch's method is used to calculate power spectral density $P(f)$.

$$Total\ power\ = \int_{0}^{0.4} P(f)\, df$$

$$High - frequency\ power\ = \int_{0.15}^{0.4} P(f)\, df$$

$$Normalized\ high - frequency\ power\ = \frac{High - frequency\ power}{Total\ power}$$

5. Cardiac vagal index (CVI) and cardiac sympathetic index (CSI):

The width (SD1) and length (SD2) of the Poincaré plot, a graphical representation of the correlation between consecutive NN intervals, are used to calculate CVI and CSI.

$$CSI\ = \frac{SD1}{SD2}$$

$$CVI\ = \ log_{10}(SD1\ *\ SD2)$$

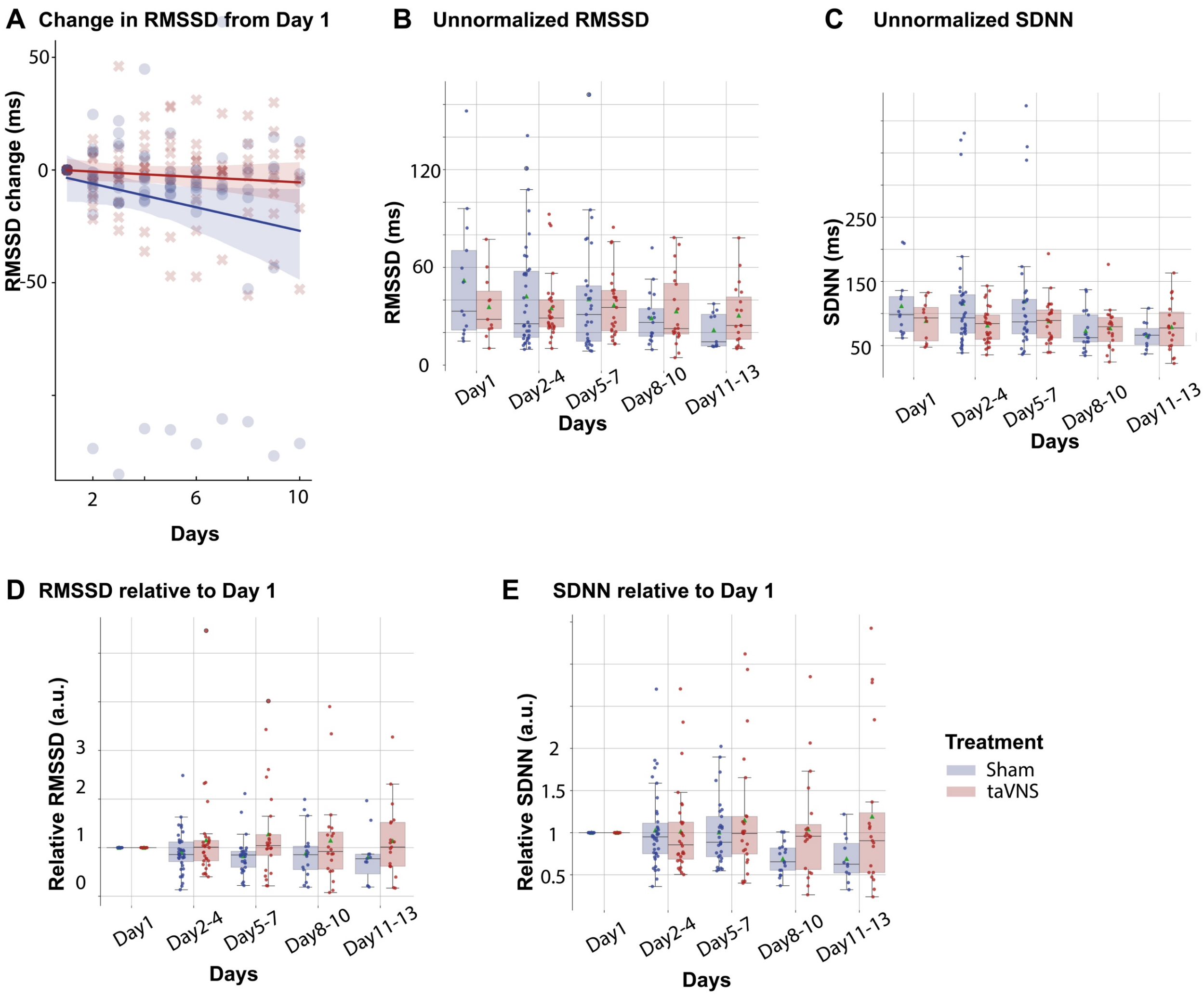

Supplementary Figure 1 (related to **Error! Reference source not found.**) **Heart rate variability in taVNS and Sham treatment groups. A.** In the Linear regression model (RMSSD change ~ Day * Treatment), the coefficient for interaction effect is 2.01 ($p = 0.21$), the coefficient for Day is -2.61 ($p=0.02$), and the coefficient for Treatment is 1.38 ($p = 0.88$). RMSSD: Root mean square of successive differences of normal RR intervals. SDNN: Standard deviation of normal RR intervals.

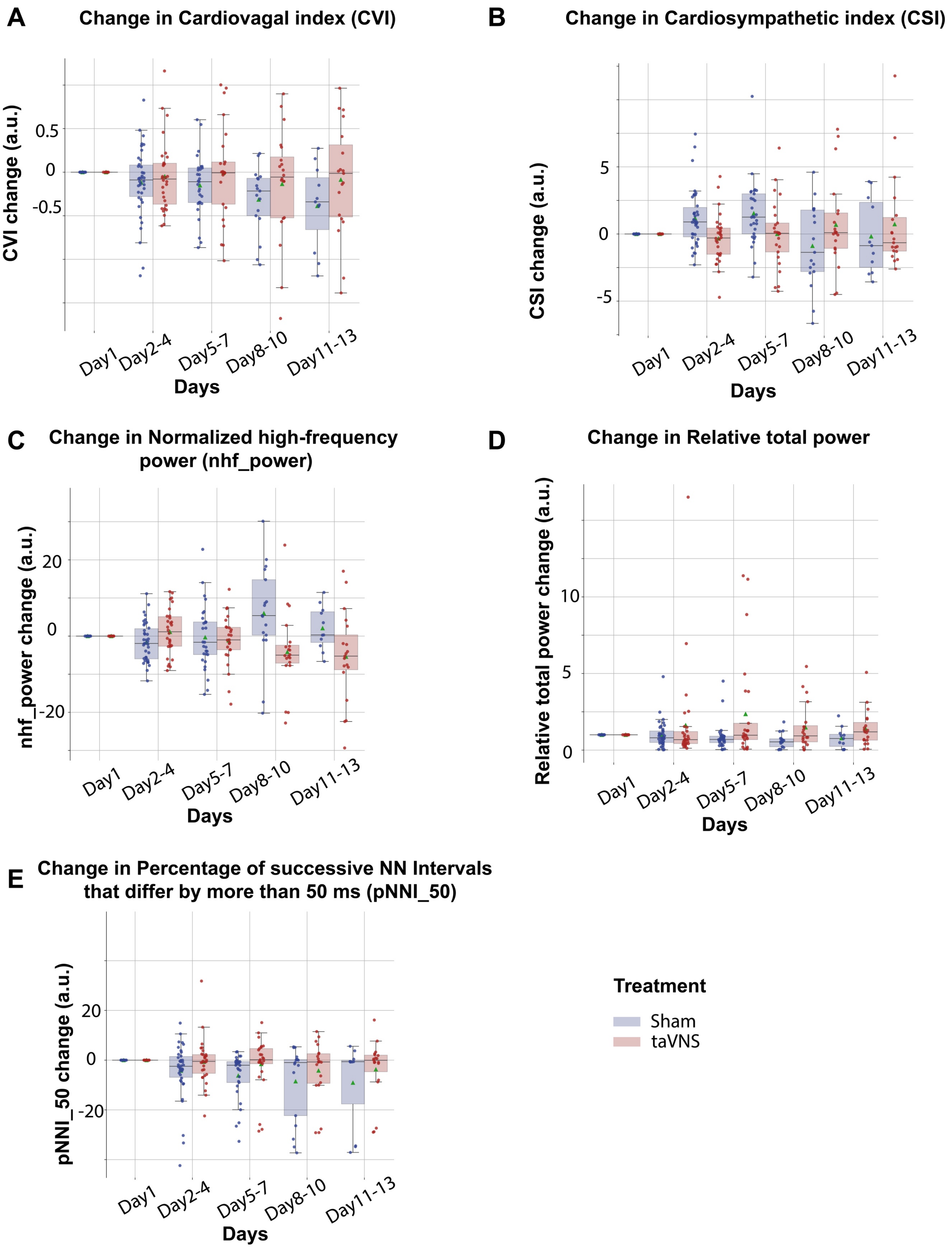
A
Change in Cardiovagal index (CVI)
CVI change (a.u.)
0.5
0
-0.5
Day1
Day2-4
Day5-7
Day8-10
Day11-13
Days
B
Change in Cardiosympathetic index (CSI)
CSI change (a.u.)
5
0
-5
Day1
Day2-4
Day5-7
Day8-10
Day11-13
Days
C
Change in Normalized high-frequency power (nhf_power)
nhf_power change (a.u.)
20
0
-20
Day1
Day2-4
Day5-7
Day8-10
Day11-13
Days
D
Change in Relative total power
Relative total power change (a.u.)
10
5
0
Day1
Day2-4
Day5-7
Day8-10
Day11-13
Days
E
Change in Percentage of successive NN Intervals that differ by more than 50 ms (pNNI_50)
pNNI_50 change (a.u.)
20
0
-20
Day1
Day2-4
Day5-7
Day8-10
Day11-13
Days
Treatment
Sham
taVNS

Supplementary Figure 2 (related to **Error! Reference source not found.**) **Time-domain and frequency-domain cardiac measures in taVNS and Sham treatment groups.** These standard heart rate variability metrics were used to perform factor analysis.

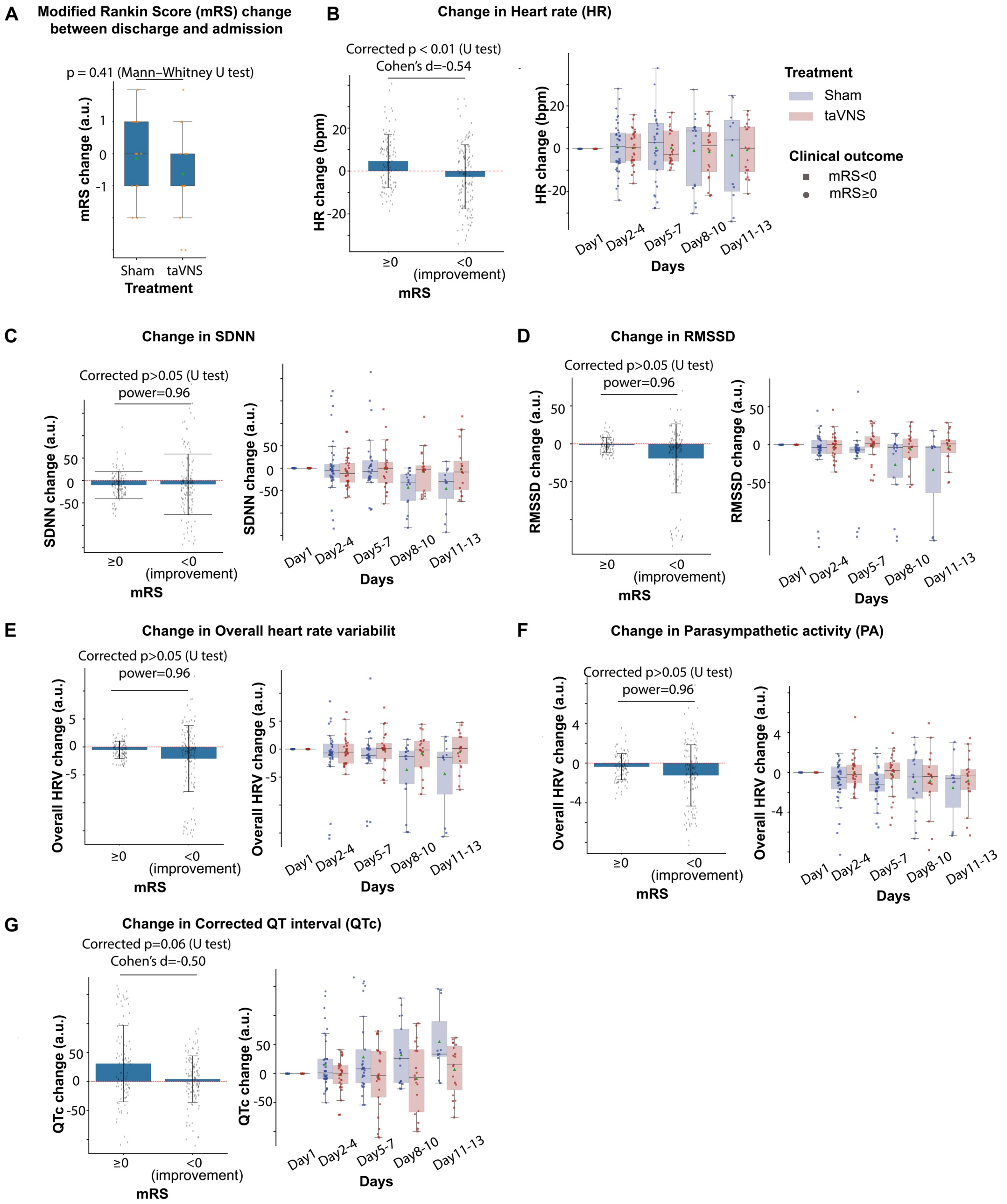

Supplementary Figure 3 (related to **Error! Reference source not found.**) **The impact of clinical outcome on heart rate variability. A**. Change in mRS change was similar between taVNS and Sham treatment groups. **B**. Change in heart rate during treatment

for both treatment groups, as compared to first hospitalized day, was lower in patients with improved mRS upon discharge (i.e., mRS change < 0). **C–F**. The relationship between improved mRS and changes in cardiovascular metrics. N(mRS < 0) = 122, N(mRS > 0) = 98.

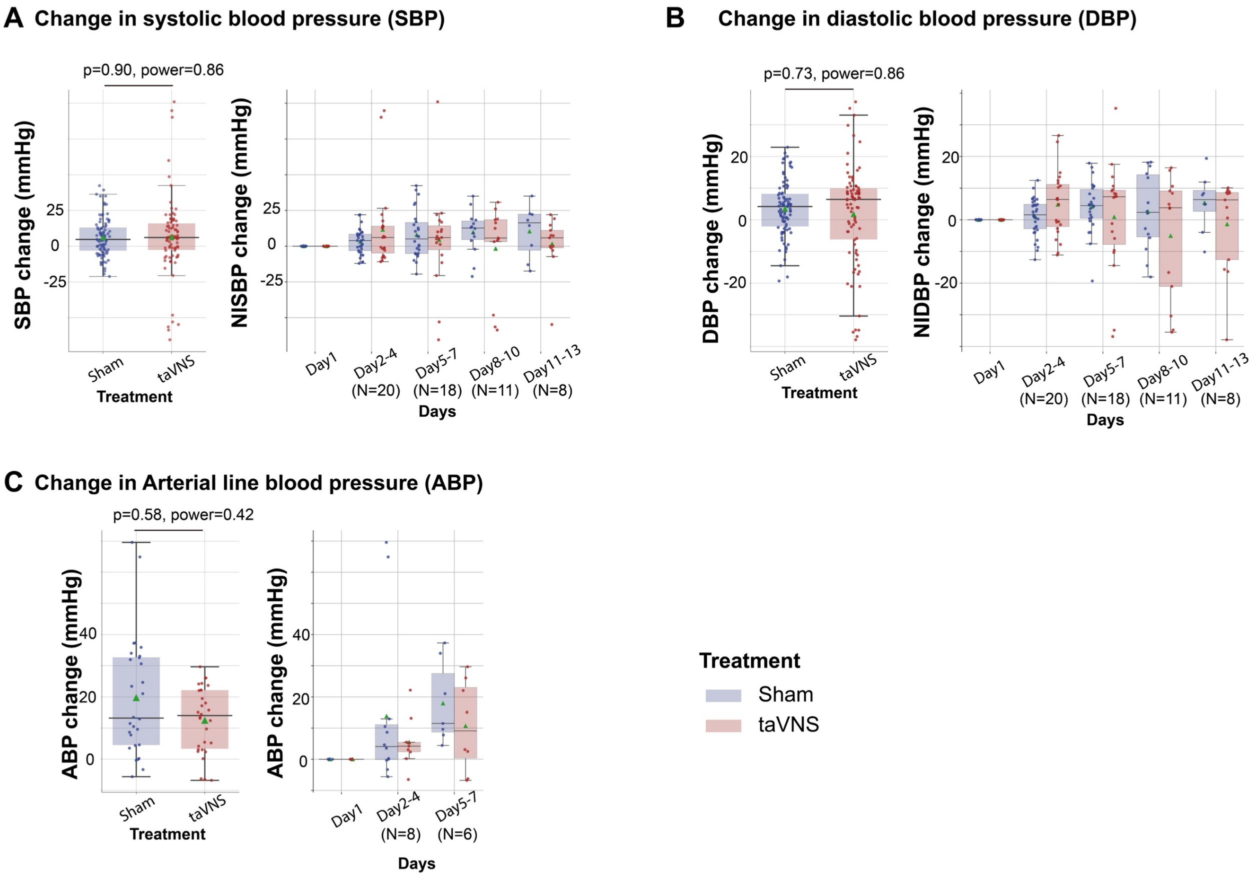

Supplementary Figure 4 (related to **Error! Reference source not found.**) **The effect of taVNS on arterial line blood pressure monitoring and noninvasive blood pressure monitoring.** Mann–Whitney U tests were used to compare changes in blood pressure between treatment groups.

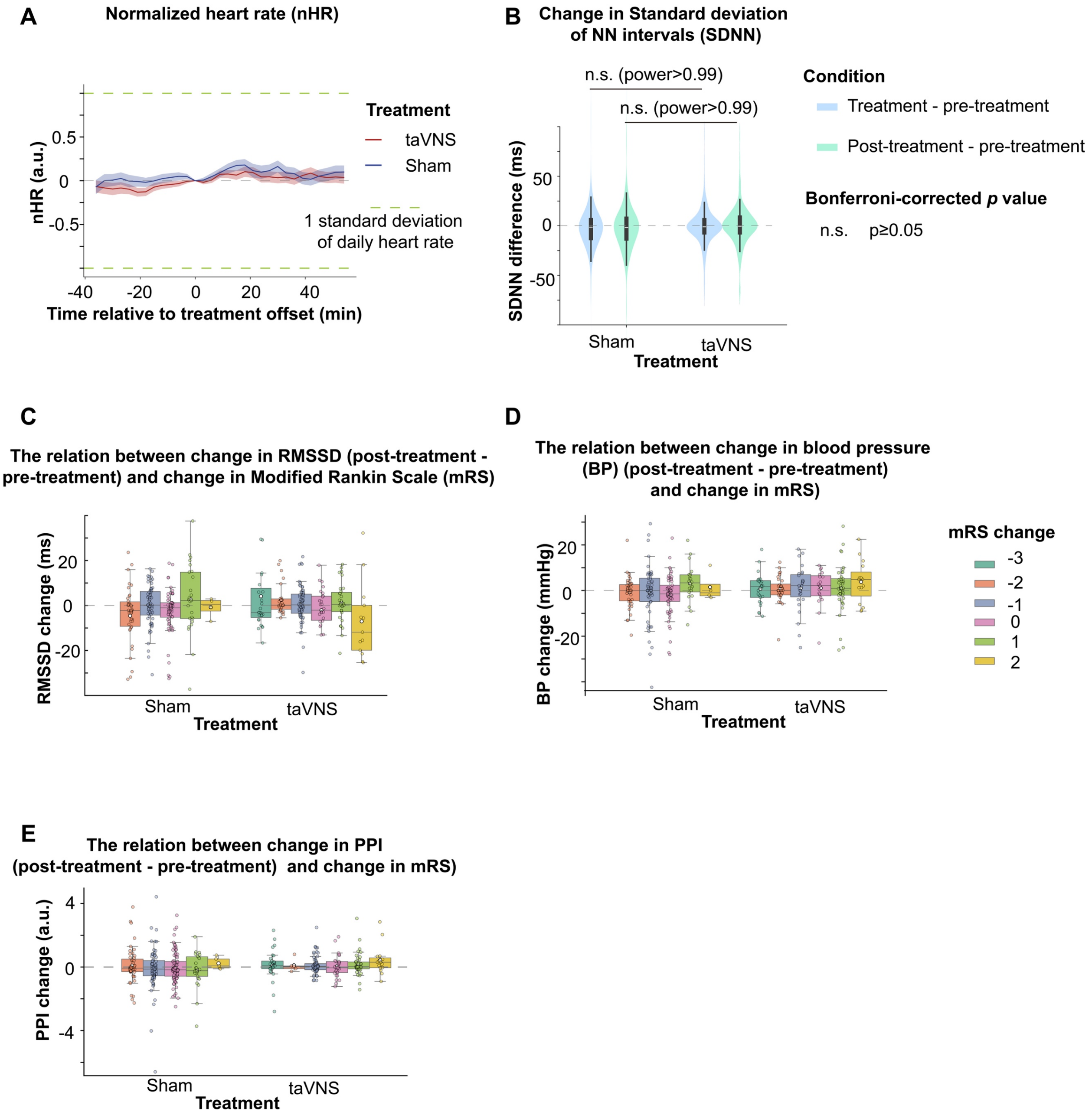

Supplementary Figure 5 (related to **Error! Reference source not found.**) **Cardiac effects of acute taVNS. A**. Temporal dynamics of normalized heart rate. Normalized heart rate for the two treatment groups at the treatment offset were set to 0 The data is presented as a mean ± standard error. **B**. Comparison of change in SDNN values from pre-treatment to treatment period (blue) and post-treatment period (green) between treatment groups. No significant differences were observed between treatment groups

based on Mann–Whitney U tests. **C-E**. The relationship between changes in the modified Rankin Scale (mRS) scores and changes in differential measures of cardiovascular parameters from pre-treatment to post-treatment periods.

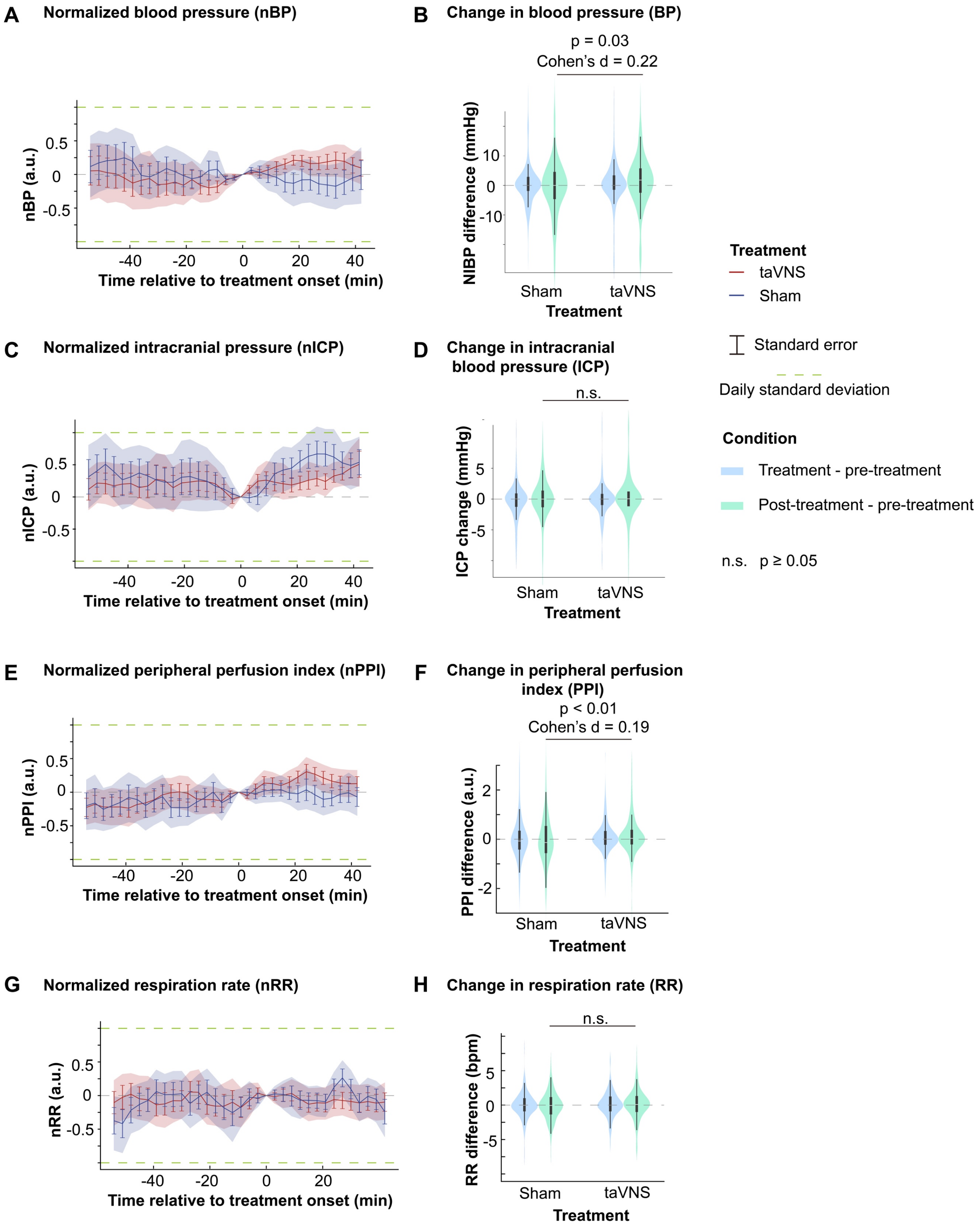

Supplementary Figure 6 (related to **Error! Reference source not found.**) **Vascular effects of acute taVNS. A, C, E, G (left)**. Temporal dynamics of normalized blood

pressure (BP), intracranial blood pressure (ICP), peripheral perfusion index (PPI), and respiration rate (RR) in both treatment groups, aligned with the treatment onset. **B, D, F, H (right).** Comparison of change in BP, ICP, PPI, and RR from pre-treatment to treatment (blue) and post-treatment (green) periods. Mann–Whitney U tests were used to compare the change in vital signs between groups. The powers are > 0.99 for RR and 0.98 for ICP.

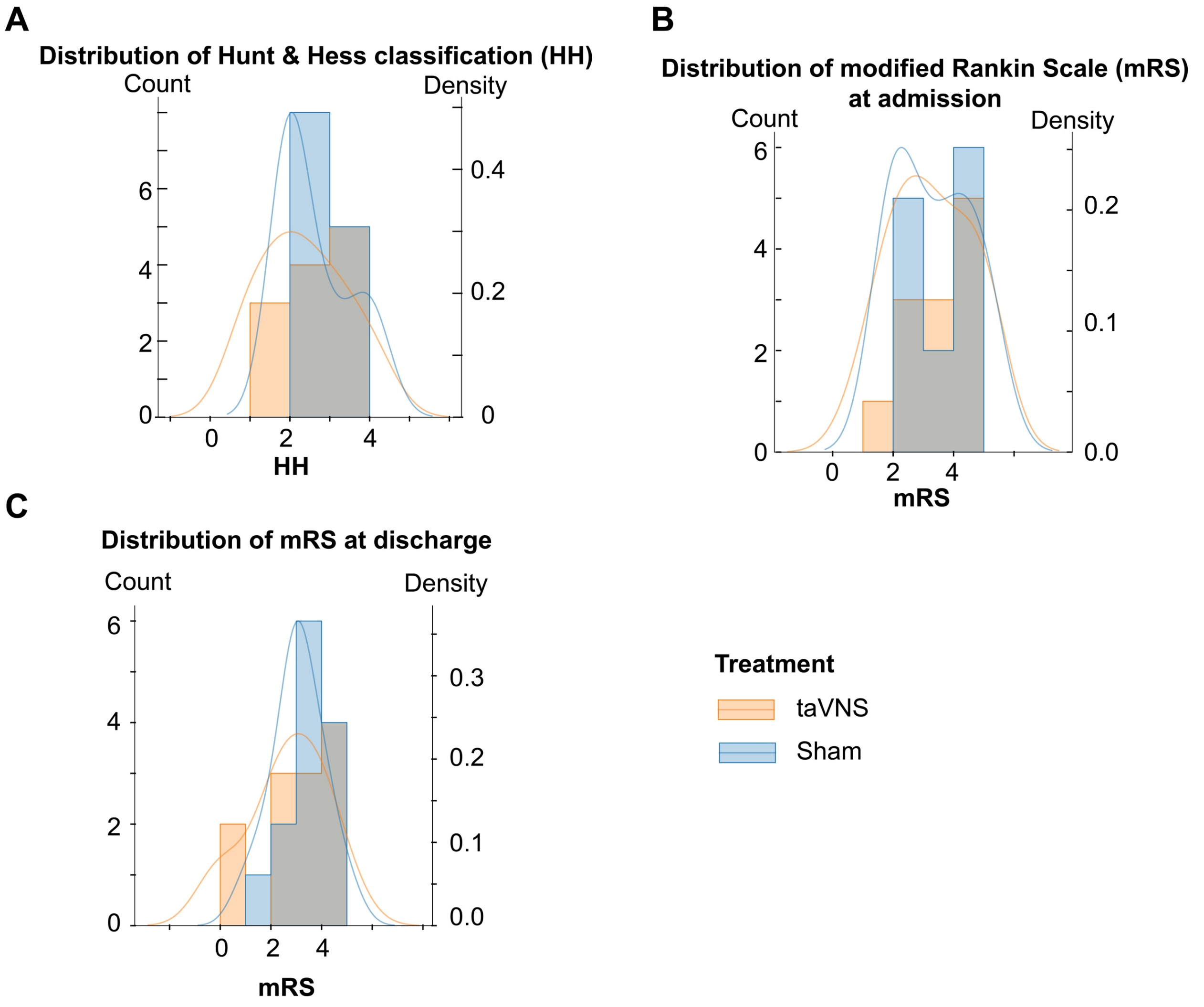

Supplementary Figure 7 The distribution of Hunt & Hess classification and modified Rankin Scale for taVNS and Sham groups.

Patient	Age	Gender	Race	HH	mRS (admission)	mRS (discharge)	Vasospasm treated by blood pressure goal augmentation
2020004	71 – 75	M	White	3	5	5	Y
2020005	51 – 55	M	Black/African American	3	4	3	N
2020006	51 – 55	F	White	2	2	3	N
2020007	61 – 65	M	White	2	3	4	Y
2020008	66 – 70	F	White	2	2	3	Y
2020009	21 – 25	F	Black/African American	1	2	2	N
2020010	71 – 75	F	White	2	3	2	N
2020011	66 – 70	M	White	4	5	4	N
2020012	61 – 65	F	White	4	5	2	N
2020013	71 – 75	F	White	2	3	0	Y
2020014	71 – 75	F	White	2	3	3	Y
2020015	41 – 45	M	Black/African American	4	5	4	Y
2020016	46 – 50	F	White	2	4	3	N
2020017	51 – 55	F	Black/African American	2	2	1	Y
2020018	81 – 85	F	White	1	3	3	Y
2020019	71 – 75	F	Black/African American	4	5	3	N
2020020	46 – 50	F	Black/African American	2	2	3	N
2020021	66 – 70	F	White	2	1	0	N
2020022	36 – 40	F	White	3	4	2	N
2020023	61 – 65	F	White	3	4	2	N
2020024	81 - 85	F	White	2	2	3	N
2020025	51 - 55	F	White	3	4	4	N
2020026	71 - 75	F	White	1	2	4	N
2020027	41 – 45	F	Black/African American	2	2	4	N
2020028	66 - 70	M	White	4	5	5	Y

Supplementary Table 1 Patient demography. HH: Hunt & Hess classification. mRS: modified Rankin Scale.

Supplementary Table 2. Summary of Statistical Tests. Bolded values were used to make inferences in this paper.

Test name	Variable	Distribution A	Distribution B	N(A)	N(B)	p	statistics	Effect size
Mann-Whitney U	RMSSD change (Day5-7)	VNS	Sham	25	29	**0.01**	U(A) = 510	0.41
Mann-Whitney U	RMSSD change (Day8-10)	VNS	Sham	20	17	0.33	U(A) = 202	0.54
Mann-Whitney U	RMSSD change (Day11-13)	VNS	Sham	19	11	0.20	U(A) = 135	0.66
Mann-Whitney U	SDNN change (Day5-7)	VNS	Sham	25	29	0.96	U(A) = 359	-0.10
Mann-Whitney U	SDNN change (Day8-10)	VNS	Sham	20	17	**0.01**	U(A) = 253	0.82
Mann-Whitney U	SDNN change (Day11-13)	VNS	Sham	19	11	0.12	U(A) = 135	0.79
Equivalence test (two one sided t-tests)	HR change	VNS	Sham	94	95	**0.006 (lower), 0.004 (upper)**	2.53 (lower) -2.72 (upper)	-0.01
Mann-Whitney U	QTc change	VNS	Sham	94	95	**<0.01**	U(A) = 3539	**-0.57**
Equivalence test (two one-sided t-tests)	QTc change margin:30ms	VNS	Sham	94	95	0.50 (lower), **1.45*** $\mathbf{10^{-13}}$ (upper)	-0.004 (lower) -7.86 (upper)	-0.57
Mann-Whitney U	Overall HRV change	VNS	Sham	94	95	**0.04**	U(A) = 5237	**0.37**
Mann-Whitney U	Parasympathetic activity change	VNS	Sham	94	95	**0.04**	U(A) = 5238	0.29
Mann-Whitney U	ICP change	VNS	Sham	66	52	0.61	U(A) = 1972	0.25
Equivalence test (two one-sided t-tests)	ICP change margin:2mmHg	VNS	Sham	66	52	**3.66*** $\mathbf{10^{-13}}$ (lower), **3.33*** $\mathbf{10^{-10}}$ (upper)	8.07 (lower) -6.73 (upper)	0.12
Mann-Whitney U	BP change	VNS	Sham	66	81	0.73	U(A) = 2842	-0.11
Equivalence test (two one-sided t-tests)	BP change margin:2mmHg	VNS	Sham	66	81	0.07(lower), 0.002(upper)	1.51 (lower) -3.00 (upper)	-0.12

Mann-Whitney U	SBP change	VNS	Sham	66	81	0.90	U(A) = 2719	-0.07
Mann-Whitney U	DBP change	VNS	Sham	66	81	0.73	U(A) = 2846	-0.17
Mann-Whitney U	ABP change	VNS	Sham	28	24	0.46	U(A) = 295	-0.48
Mann-Whitney U	PPI change	VNS	Sham	83	95	**0.002**	U(A) = 2877	-0.49
Mann-Whitney U	RR change	VNS	Sham	94	95	**0.004**	U(A) = 5530	0.37
Mann-Whitney U	HR difference (post-pre)	VNS	Sham	188	199	0.28	U(A) = 17527	0.10
Wilcoxon signed rank	HR difference (post-pre)	VNS		188		**0.02**	7525	0.11
Mann-Whitney U	QTc difference (post-pre)	VNS	Sham	188	198	0.86	U(A) = 18412	0.02
Mann-Whitney U	RMSSD difference (post-pre)	VNS	Sham	188	199	0.31	U(A) = 17581	0.14
Mann-Whitney U	SDNN difference (post-pre)	VNS	Sham	188	199	0.48	U(A) = 17923	0.03
Mann-Whitney U	HR difference(post-pre)	mRS change<-1	mRS change≥ -1	53	122	**0.01**	U(A) = 4019	0.38
Mann-Whitney U	BP difference(post-pre)	VNS	Sham	159	180	**0.03**	U(B) = 12400	0.21
Mann-Whitney U	ICP difference(post-pre)	VNS	Sham	146	114	0.82	U(B) = 12400	0.09
Mann-Whitney U	PPI difference(post-pre)	VNS	Sham	186	227	**0.002**	U(B) = 17386	0.19
Mann-Whitney U	RR difference(post-pre)	VNS	Sham	214	224	0.10	U(B) = 21806	0.11
